# Supplementary material for: The Photocatalytic Degradation of Enrofloxacin Using an Ecofriendly Natural Iron Mineral: The Relationship Between the Degradation Routes, Generated Byproducts, and Antimicrobial Activity of Treated Solutions
Source: Molecules. 2024 Dec 18;29(24):5982. doi: 10.3390/molecules29245982 (PMC11676763; doi:10.3390/molecules29245982)
Supplement: Supplementary file 1 [file molecules-29-05982-s001.zip › molecules-3286283-supplementary.pdf]

Supplementary information

The Photocatalytic Degradation of Enrofloxacin Using an Ecofriendly Natural Iron Mineral: The Relationship Between the Degradation Routes, Generated Byproducts, and Antimi-crobial Activity of Treated Solutions .

Supplementary figures

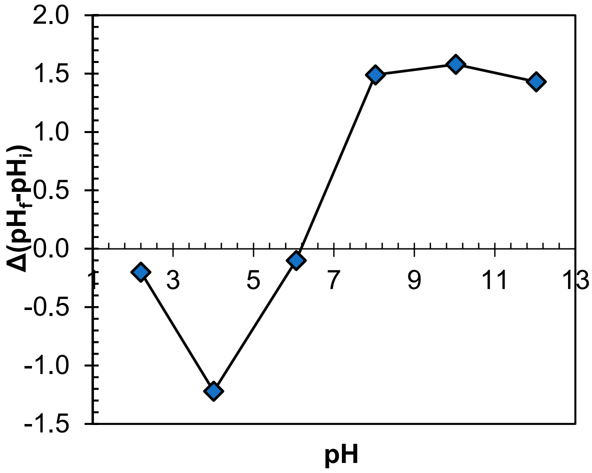

(a)

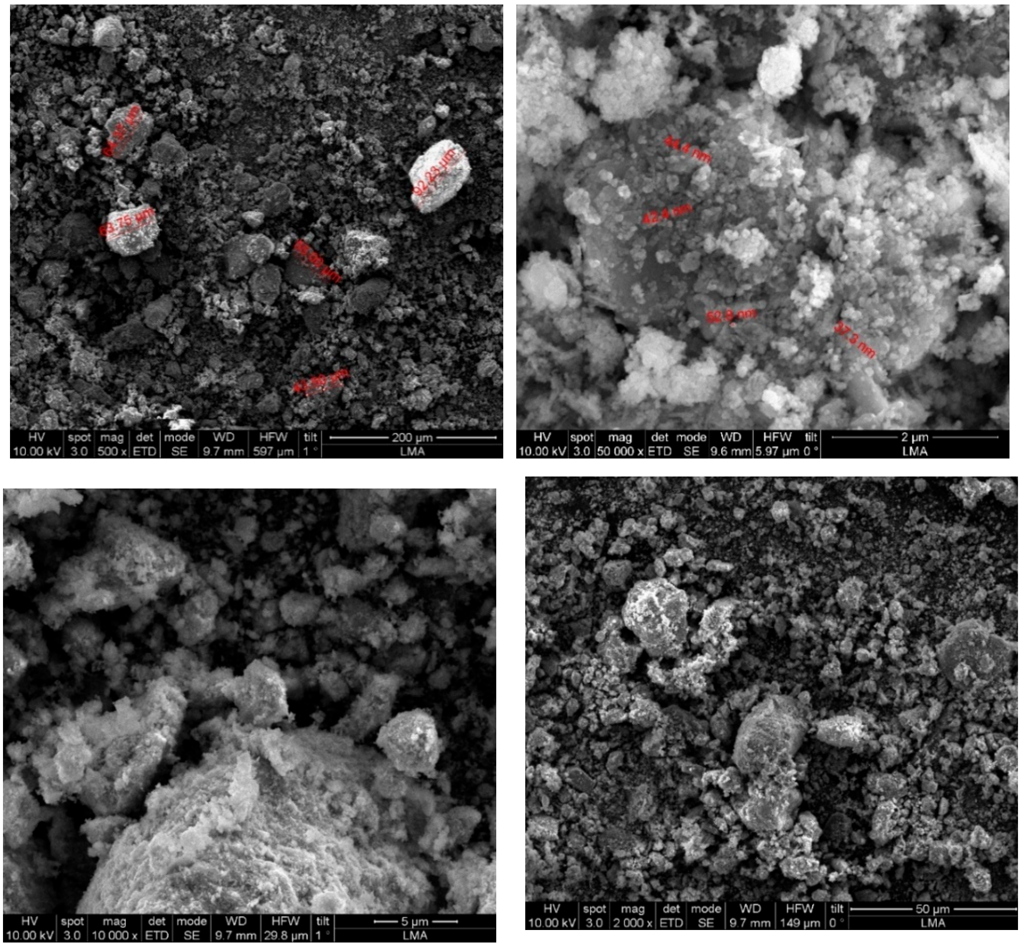

(b)

Figure S1. Characterization of NIM. **(a):** Point of zero charge (PZC). **(b)**: SEM analyses at different levels of magnification

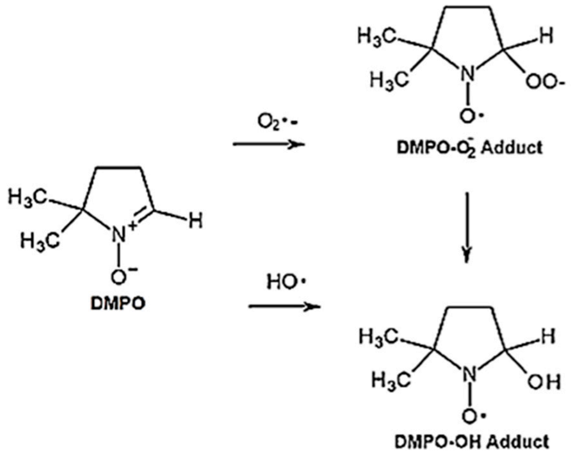

Figure S2. DMPO transformation to DMPO-OH and DMPO-OOH adducts [1–3]

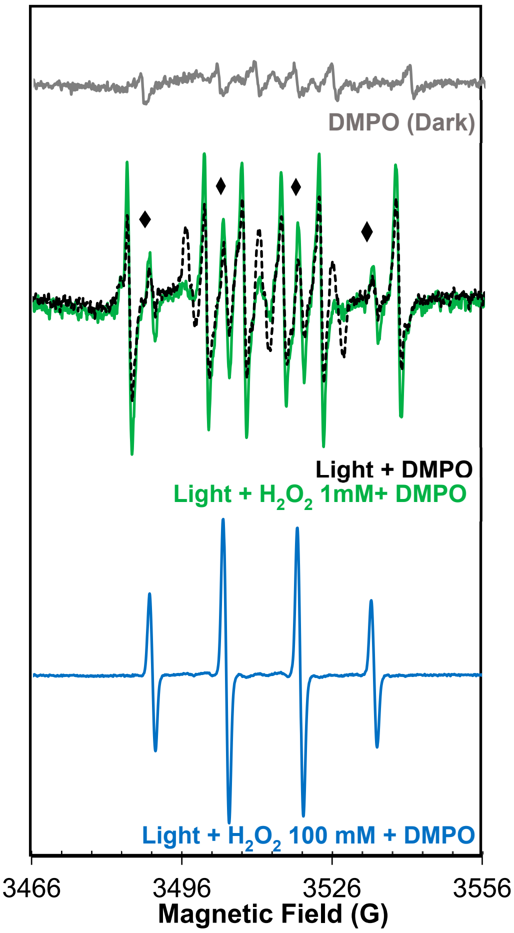

Figure S3. EPR spectrum of radicals captured H_2O_2-Light system by DMPO in water.

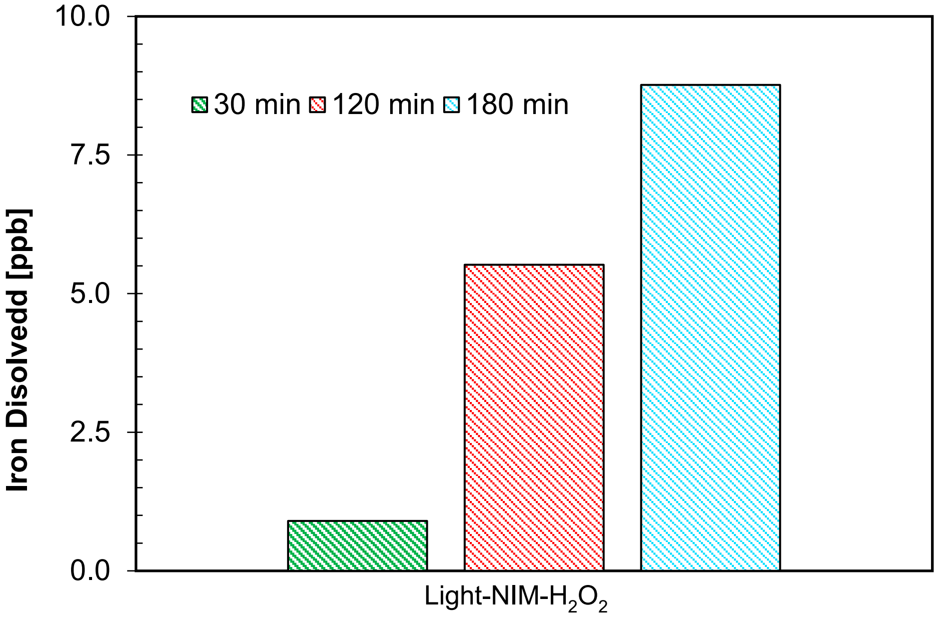

Figure S4. Dissolved iron ions during ENR degradation under Light-NIM-H_2O_2 process

Supplementary Tables

Table S1. Characteristics of the identified by-products of enrofloxacin (ENR).

**Compound**	**Structure Proposed**	**Chemical Name**	**[M+H]+**	**Main Fragments [M+H]+**	**Also found in refs**
**ENR**	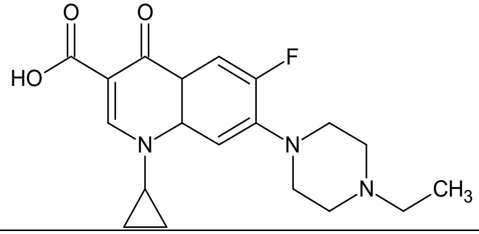	1-cyclopropyl-7-(4-ethylpiperazin-1-yl)-6-fluoro-4-oxo-1,4,4a,8a-tetrahydroquinoline-3-carboxylic acid	[360]		
**P1**	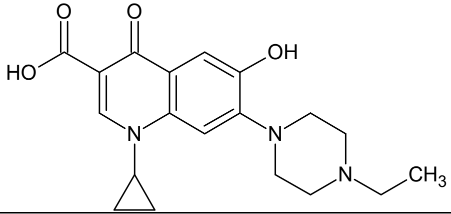	1-cyclopropyl-7-(4-ethylpiperazin-1-yl)-6-hydroxy-4-oxo-1,4-dihydroquinoline-3-carboxylic acid	[358]	329, 310, 289, 279, 263, 259, 234, 213.	[4][5]
**P2**	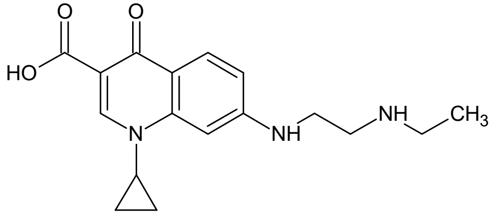	1-cyclopropyl-7-{[2-(ethylamino)ethyl]amino}-4-oxo-1,4-dihydroquinoline-3-carboxylic acid	[316]	279, 258, 229.	[4][5]

Compound	Structure Proposed	Chemical Name	$[M+H]^+$	Main Fragments $[M+H]^+$	Also found By
**P3**	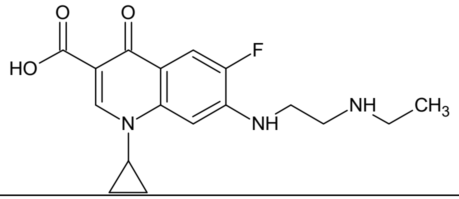	7-[(2-aminoethyl) (ethyl)amino]-1-cyclopropyl-6-fluoro-4-oxo-1,4-dihydroquinoline-3-carboxylic acid	[334]	320,301,<br>278,259,<br>213	[5]
**P4**<br>**(CIP)**	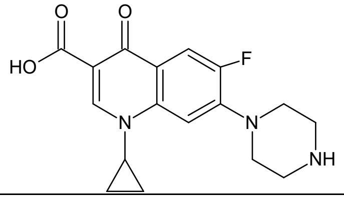	1-cyclopropyl-6-fluoro-4-oxo-7-(piperazin-1-yl)-1,4-dihydroquinoline-3-carboxylic acid	[332]	278, 213	[6][7][8]
**P5**	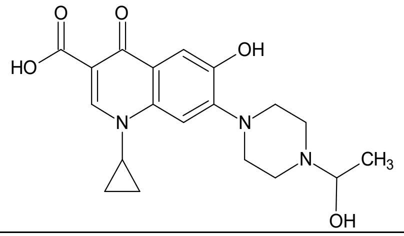	1-cyclopropyl-6-hydroxy-7-[4-(1-hydroxyethyl)piperazin-1-yl]-4-oxo-1,4-dihydroquinoline-3-carboxylic acid	[374]	375,279, 259, 229, 213	-
**P6**	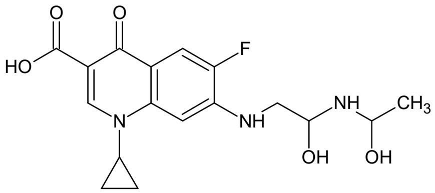	1-cyclopropyl-6-fluoro-7-[(hydroxymethyl){2-[(hydroxymethyl)amino]ethyl}amino]-4-oxo-1,4-dihydroquinoline-3-carboxylic acid.	[366]	242, 224, 207, 194, 166	

**Compound**	**Structure Proposed**	**Chemical Name**	**$[M+H]^+$**	**Main Fragments $[M+H]^+$**	**Also found By**
**P7**	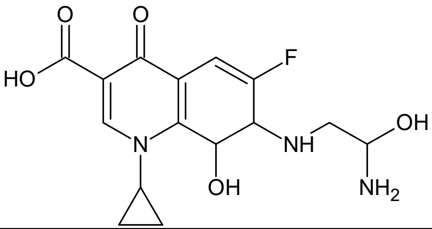	7-[(2-amino-1-hydroxy-ethyl)amino]-1-cyclopropyl-6-fluoro-8-hydroxy-4-oxo-1,4,7,8-tetrahydroquinoline-3-carboxylic acid	[340]	332, 166	
**P8**	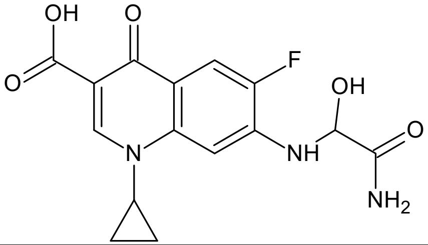	1-cyclopropyl-6-fluoro-8-hydroxy-7-[(2-nitrosoethyl)amino]-4-oxo-1,4-dihydroquinoline-3-carboxylic acid	[336]	332, 352	
**P9**	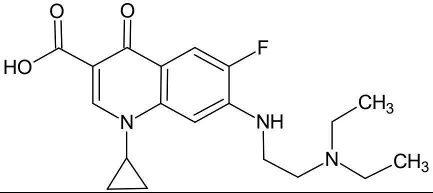	1-cyclopropyl-7-{[2-(diethylamino)ethyl]amino}-6-fluoro-4-oxo-1,4-dihydroquinoline-3-carboxylic acid	[362]	329,296, 284,279, 241, 213, 204, 167, 117, 114	
**P10**	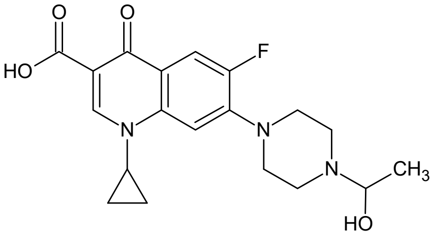	1-cyclopropyl-6-fluoro-7-[4-(2-hydroxyethyl)piperazin-1-yl]-4-oxo-1,4-dihydroquinoline-3-carboxylic acid	[376]	340, 279, 258, 240, 212	

Table S2. Main characteristics of the effluents of municipal wastewater treatment plant (EWWTP).

Property	Unit	Value Sample 2	Analytical method
**pH**		7.2	(SM 4500 HB) [9]
**Conductivity**	µS cm^{-1}	2156	27888:1994 Asociación Española de Normalización y Certificación (AENOR) [10]
**Chemical oxygen demand (COD)**	mg L^{-1}	62	(EPA 410.4)[11]
**Turbidity**	TNU	7	7027:2001 (ISO, AENOR, 2001) [12,13]
**Total Organic Carbon (TOC)**	mg L^{-1}	32	5310B (Eaton et al., 2005) [14]
**Dissolved oxygen**	mg L^{-1}	6.2	Método Winkler, 4500 O-C (Eaton et al., 2005) [14][13]

References Suplementary Information

1. Hawkins, C.L.; Davies, M.J. Detection and Characterisation of Radicals in Biological Materials Using EPR Methodology. *Biochim Biophys Acta Gen Subj* 2014, *1840*, 708–721.
2. Dvoranová, D.; Barbieriková, Z.; Brezová, V. Radical Intermediates in Photoinduced Reactions on TiO2 (An EPR Spin Trapping Study). *Molecules* **2014**, *19*, 17279–17304, doi:10.3390/molecules191117279.
3. Villamena, F.A.; Merle, J.K.; Hadad, C.M.; Zweier, J.L. Superoxide Radical Anion Adduct of 5,5-Dimethyl-1-Pyrroline N-Oxide (DMPO). 2. The Thermodynamics of Decay and EPR Spectral Properties. *Journal of Physical Chemistry A* **2005**, *109*, 6089–6098, doi:10.1021/jp0524330.
4. Wammer, K.H.; Korte, A.R.; Lundeen, R. a.; Sundberg, J.E.; McNeill, K.; Arnold, W. a. Direct Photochemistry of Three Fluoroquinolone Antibacterials: Norfloxacin, Ofloxacin, and Enrofloxacin. *Water Res* **2013**, *47*, 439–448, doi:10.1016/j.watres.2012.10.025.
5. Sturini, M.; Speltini, A.; Maraschi, F.; Profumo, A.; Pretali, L.; Fasani, E.; Albini, A. Photochemical Degradation of Marbofloxacin and Enrofloxacin in Natural Waters. *Environ Sci Technol* **2010**, *44*, 4564–4569, doi:10.1021/es100278n.
6. Ge, L.; Chen, J.; Wei, X.; Zhang, S.; Qiao, X.; Cai, X.; Xie, Q. Aquatic Photochemistry of Fluoroquinolone Antibiotics: Kinetics, Pathways, and Multivariate Effects of Main Water Constituents. *Environ Sci Technol* **2010**, *44*, 2400–2405, doi:10.1021/es902852v.
7. Li, Y.; Niu, J.; Wang, W. Photolysis of Enrofloxacin in Aqueous Systems under Simulated Sunlight Irradiation: Kinetics, Mechanism and Toxicity of Photolysis Products. *Chemosphere* **2011**, *85*, 892–897, doi:10.1016/j.chemosphere.2011.07.008.
8. Zhang, H.; Huang, C.H. Oxidative Transformation of Fluoroquinolone Antibacterial Agents and Structurally Related Amines by Manganese Oxide. *Environ Sci Technol* **2005**, *39*, 4474–4483, doi:10.1021/es048166d.
9. Gilcreas, F.W. Future of Standard Methods for the Examination of Water and Wastewater. *Health Lab Sci* **1967**, *4*, 137–141, doi:10.1520/E0536-16.2.
10. Federation, W.E. Standard Methods for the Examination of Water and Wastewater. *American Public Health Association, American Water Works Association, Water Environment Federation* 1999, 1–6.
11. Epa Method 410.4 the Determination of Chemical Oxygen Demand By Semi-Automated Colorimetry. **1993**, 1–12.
12. UNE-EN ISO 7027 Calidad Del Agua Determinación DeLaTurbiedad. In *UNE-EN ISO 7027*; 2001.
13. American Public Health Association *Standard Methods for the Examination of Water and Wastewater*; 21st ed.; 2005;
14. Eaton Clesceri L.S. Rice E.W. and Greenberg A.E. (2005), A.D. *Standard Methods for the Examination of Water and Wastewater*; 2005; Vol. 51; ISBN 0875532357.
